# Mental health in young mothers, single mothers and their children

**DOI:** 10.1186/s12888-019-2082-y

**Published:** 2019-04-11

**Authors:** Sara Agnafors, Marie Bladh, Carl Göran Svedin, Gunilla Sydsjö

**Affiliations:** 1 Department of Clinical and Experimental Medicine, Division of Children’s and Women’s health, Linköping University, SE-581 83 Linköping, Sweden; 2 Department of Clinical and Experimental Medicine, Barnafrid, Linköping University, SE-581 83 Linköping, Sweden

**Keywords:** Maternal mental health, Young mothers, Single mothers, Child mental health

## Abstract

**Background:**

Parenthood is a life transition that can be especially demanding for vulnerable individuals. Young maternal age and maternal single status have been reported to increase the risk for adverse outcomes for both mother and child. The aim of this study was to investigate the effect of young maternal age and maternal single status on maternal and child mental health and child development at age 3.

**Methods:**

A birth-cohort of 1723 mothers and their children were followed from birth to age 3. Sixty-one mothers (3.5%) were age 20 or younger, and 65 (4.0%) reported single status at childbirth. The mothers filled out standardized instruments and medical information was retrieved from the standardized clinical assessment of the children at Child Welfare Centers, (CWC).

**Results:**

Young maternal age was associated with symptoms of postpartum depression whereas single status was not. Young mothers were more prone to report internalizing and externalizing problems in their children, while there was no association between single status and child behavioral problems. No differences were seen on child development (CWC scores). School drop-out was, however, a more influential factor on depressive symptoms postpartum than maternal age.

**Conclusion:**

Young mothers are at increased risk for symptoms of postpartum depression which indicates the need for attention in pre- and postnatal health care programs. Single mothers and their children were not found to be at increased risk for adverse outcomes. The importance of schooling was demonstrated, indicating the need for societal support to encourage adolescents to remain in school.

## Background

Becoming a parent is a large life transition that bring new challenges. Childbirth during adolescence is demanding, as it takes place during an intense mental and physical developmental stage, challenging or forcing the transition from childhood to adulthood. Single-mother families often face structural disadvantages due to having lower income and less time together with their children. Maternal vulnerability may influence not only the mental health and wellbeing of the mother herself, but also the development and wellbeing of her child [1].

Early motherhood has been shown to be associated with adverse developmental outcomes for both mothers and children [2]. Teenage mothers are at increased risk for depression [3]. The fact that adolescent mothers are still in a developmental stage may lead to difficulties when raising a child, influencing both maternal and child wellbeing. However, it has been argued that it is not the young age itself but rather associated factors such as dysfunctional relations and socioeconomic factors that predispose to adolescent pregnancy, factors that in turn add to the combination of risk factors for young mothers and their children [4]. Children of teenage mothers have been shown to have delays in cognitive and language abilities [5, 6] however, age appropriate development has been noted in smaller studies [7, 8].

The risk for psychopathology in single mothers and their children is increased, but the mechanisms for this are not known. In a Swedish study, children of single parent households (90% women) were found to be at increased risk for childhood psychopathology, suicide attempts and drug addiction [9]. As noted earlier single mothers may face not only the non-shared care of a child but also economic problems resulting from discriminatory wage levels and the absence of a second income from a partner [10, 11]. Moreover, negative parenting behaviors have been shown to be more common in single-mother households [12]. The increased risk for mental health problems in children of single-mother households has also been attributed to increased experience of stressful life events [13]. While these factors add to the total burden of risk, single motherhood stays significantly associated with youth psychopathology after controlling for poverty and maternal psychopathology [14]. However, research on maternal and child outcomes of single motherhood has mainly focused on divorced families, where the divorce itself can impact child wellbeing. Less is known about wellbeing of mothers who are single at the time of childbirth, and this is also true as concerns the health of their children. To our knowledge, cognitive development in pre-school children of single mother families has not yet been thoroughly studied.

Single as well as early motherhood has been shown to be associated with low socioeconomic status (SES) [10, 15]. Child health and development is strongly related to family SES, and researchers suggest a reciprocal relationship between these factors [15, 16]. Crosier and colleagues (1994) showed that as much as 94% of the association between single mother status and poor mental health (for the mothers) was explained by sociodemographic factors. While low SES and poor health may be especially detrimental during the first years in life due to the intense brain development, effects in terms of cognitive ability, mental health and achievement may become more apparent in older children.

There is a lack of studies examining mental health in women who are single at childbirth. The present study adds to the field by 1) examining mental health in women who are single at childbirth and 2) including measures of mental health in both mothers and their children, and also objective measurement of child development.

### Aim

The aim of the present study was to investigate the impact of early motherhood and single motherhood on maternal mental health and child wellbeing and development.

## Methods

### Subjects

In the South East Sweden Birth Cohort study (SESBiC), all mothers of children born between May 1st 1995 and December 31st 1996 in five municipalities in southern Sweden were asked to take part. In total, mothers of 1723 children  (88%) agreed to participate. The mean age of the mothers was 28.2 ± 4.6 years at the time they gave birth, and 61 mothers (3.5%) were age 20 or younger. The vast majority (96,0%, *n* = 1574) were cohabitating, 3.5% (*n* = 57) were single parents, and 0.5% (*n* = 8) reported other family arrangements. Most mothers had been born in Sweden (*n* = 1482, 88.6%), but 6.2% (*n* = 103) had been born in Europe, and 5.3% (*n* = 88) outside Europe. Of the newborn children, 52.8% were boys, and there were 27 twin pairs. The first index group consisted of the 61 mothers who were 20 years old or younger when the child was born, and was compared to all women age 21 or older at the time of childbirth. The second index group constituted of the 65 women who reported single status or other family arrangements at the time of childbirth; they were compared to women who were cohabitating at the time of childbirth. Four of the women age 20 or younger also reported other family arrangements, and one woman, age 20 or younger reported single status. At the 3-year follow-up, one child was deceased and mothers of 1452 children (84,3%) agreed to participate. In the index groups, 42 (68.9%) women who were 20 years or younger at childbirth, and 55 (84.6%) mothers who reported single status at childbirth took part. The general completion of the questionnaires was considered to be good as the partial missing data ranged between 0.1 and 5.7%.

### Procedure

The baseline assessments were carried out at Child Welfare Centers, (CWC) in the catchment area in connection with the routine check-up at the child’s age 3 months. Information about the study was given to the mothers by the CWC staff. The mothers were interviewed by a psychologist in order to evaluate psychosocial aspects of family life, and they also filled out standardized instruments. The follow-up was done at the time of the routine examination at the CWC of the children when they were 3 years old. Mothers were asked to fill out questionnaires and information was retrieved from the medical journals containing the standardized clinical assessment performed by the nurse and doctor. The controls at the CWCs’ are part of the national child welfare program offered to all children living in Sweden, and more than 95% of the families take part.

### Instruments

#### Baseline mother assessment

The Edinburgh Postnatal Depression Scale (EPDS) [17] is a self-report questionnaire designed to screen for postnatal depression in community samples. It contains 10 items, each with a scale 0–3 with a total score of 30. The EPDS is concerned with symptoms experienced during the preceding week and was filled out by the mothers at baseline. A cut-off level of 9/10 has previously been shown to render a sensitivity of 96% and specificity of 62% for Major Depressive Disorder (MDD) [18]. A cut-off level of 10 was thus used in the present study as was done previously for screening purposes [19].

#### Follow-up child assessment

The Child Behaviour Check List/2–3 (CBCL) [20] is an extensively used form assessing child behavior during the preceding two months in the two main domains of internalizing and externalizing problems. It holds 100 items, scored 0–2 from “not a problem” to “often a problem”. The CBCL has been used worldwide, and more specifically also in Scandinavian population based studies, and has proven to be an effective screening tool for child psychiatric disorders [21]. The Swedish version of the CBCL 2/3 was filled out by the mothers at the 3-year follow-up. The 90th percentile was set as a cut-off.

### Medical data

#### CWC child assessment at follow-up

The CWC assessment was based on the data recorded after the standardized clinical examination by a nurse and pediatrician made when the child was 3 years old. All children living in Sweden are offered regular health controls in order to detect developmental delays or medical or behavioral symptomatology at an early stage. In each of the following domains a score was set: parental interview, observation, language/communication, gross/fine motor skills, general cooperation, general social maturity and general development. Each domain was scored “without remark”, “uncertain” and “abnormal with/without referral”. Without remark was scored 0, uncertain 1, abnormal without referral 2 and with referral 3. In the analysis, children with a score of ≥3 were compared to children with a score of 2 and under. The reliability of the CWC score index was acceptable (alpha coefficient 0.74).

### Data analysis

Initially, Chi^2^ was used to examine the difference between risk groups and non-risk groups. The two groups (young mothers and single mothers) were modelled separately. Multivariate analyses, with EPDS, CBCL scales and CWC score as dependent variables and maternal age at childbirth, cohabitation as well as sociodemographic covariates (unemployment, education and profession), were performed using logistic regression. The multivariate analysis consisted of conditional stepwise logistic regression considering full factorial models. Results are presented with corresponding Odds Ratios (OR) and 95% Confidence Intervals (CI). A *p*-value < 0.05 (two-sided) was considered statistically significant. All statistical analyses were performed using IBM SPSS version 24 (IBM Corporation, Armonk, NY).

### Drop-out rate analysis

The total dropout rate was 34.5% (*n* = 581). Of mothers age 20 or younger, 68.9% (*n* = 42) took part in the follow-up compared to 84.1% (1397) of mothers age 21 and above (*p* = 0.002). No significant differences were found in participation rates between mothers who reported single status at childbirth 84.6% (*n* = 55) and cohabitating mothers 83.4% (*n* = 1313). Likewise, no differences were found between participants and non-participants regarding symptoms of postpartum depression. When immigrant status was compared between participants and non-participants, significant differences were found; 66.3% (*n* = 987) of mothers born in Sweden (*n* = 1489) took part compared to 55.9% (*n* = 57) born in Europe (*n* = 102) and 46.0% (*n* = 40) born outside Europe (*n* = 87), (*p* < 0.001).

## Results

### Prevalence

Frequency characteristics of the study population are shown in Table 1 (Table 1). There was no significant correlation between being a young mother and being a single mother at the time of childbirth (Pearsons *r* = 0.047, *p* = 0.058), however, it has to be taken into consideration that the groups were small and the *p*-value was close to 0.05.

**Table 1:** Frequency characteristics of the study population

Variable	*N* (%)	Median/Range or M/SD
Independent Variables		
Maternal age at childbirth (M/SD)	1723	28.2/4.6
≤ 20	61 (3.5%)	
Cohabitation status at child birth	1639	
Single or other arrangements	65 (4.0%)	
Maternal origin	1678	
Outside Europe	87 (5.2%)	
Maternal education at baseline	1626	
No compulsory/upper secondary	157 (9.7%)	
Maternal unemployment at baseline	1623	
Mother unemployed	115 (7.1%)	
Parental unemployment 3 years	1430	
One or both parents unemployed	184 (12.9%)	
Parental profession 3 years	1390	
Blue collar/unemployed	328 (23.6%)	
Dependent Variables		
EPDS	1679	4/0–23
≥ 10	204 (12.0%)	
CBCL Internalizing	1428	3/0–23
≥ 90th percentile	136 (7.9%)	
CBCL Externalizing	1428	7/0–34
≥ 90th percentile	134 (9.4%)	
CWC Score	1388	0/0–13
≥ 3	95 (6.8%)	

Note: *EPDS* Edinburgh Postnatal Depression Scale, *CBCL* Child Behaviour Checklist, *CWC* Child Welfare Center

### Young mothers

#### Crosstabs

Among mothers age 20 or younger at childbirth, 23.0% reported symptoms of postpartum depression, compared to 11.6% of women age 21 and older (*p* = 0.008). At the 3-year follow-up, 21.4% reported externalizing problems in their children compared to 9.0% of mothers age 21 and above (*p* = 0.007). Likewise, 21.4% of mothers age 20 or younger, reported internalizing problems in their children, compared to 9.2% of mothers age 21 or older at child birth (*p* = 0.008). There were no significant differences in children’s CWC scores for young mothers.

#### Logistic regression

In multivariate logistic regression, mothers age 20 or younger were more than twice as likely to develop symptoms of postpartum depression compared to mothers age 21 or older (OR 2.222, CI 1.151–4.290) (Table 2). Regarding behavioral problems, young mothers were more prone to report externalizing and internalizing problems in their children at age 3 (OR 2.253, CI 1.005–5.050; OR 2.381, CI 1.065–5.324). No association was found between young maternal age and CWC scores at age 3. However, lack of completion of compulsory or upper secondary school was more strongly associated with symptoms of postpartum depression than maternal age itself (OR 3.276, CI 1.151–4.290). Parental unemployment had a highly significant impact on all scales, including CWC score (OR 3.079, CI 1.784–5.315).

**Table 2:** Odds ratios for women age ≤ 20 at childbirth, when predicting depressive symptoms as well as behavior and development in their children at age 3

	Odds Ratio	95.0% CI for OR	*p*-value
EPDS ≥ 10			
Maternal age ≤ 20	2.222	1.151–4.290	0.017
Maternal education	3.276	2.208–4.860	< 0.001
Maternal unemployment	1.676	1.015–2.768	0.044
Externalizing ≥ 90th percentile			
Maternal age ≤ 20	2.253	1.005–5.050	0.049
Parental unemployment	2.810	1.790–4.412	< 0.001
Parental profession	1.815	1.207–2.729	0.004
Internalizing ≥ 90th percentile			
Maternal age ≤ 20	2.381	1.065–5.324	0.035
Parental unemployment	2.446	1.540–3.885	< 0.001
Parental profession	1.962	1.304–2.954	0.001
CWC Score ≥ 3			
Maternal age ≤ 20	0.628	0.144–2.747	0.537
Parental unemployment	3.079	1.784–5.315	< 0.001
Parental profession	1.769	1.067–2.932	0.027

Note: *CI* Confidence Interval, *OR* Odds ratio, *EPDS* Edinburgh Postnatal Depression Scale, *CWC* Child Welfare Center. Logistic regression. Dependent variables: EPDS, CBCL (internalizing, and externalizing), CWC score. Independent variables (0 is used as a reference level): Maternal age at childbirth (0 ≥ 21 years of age, 1 ≤ 20 years of age), Maternal education (0 = accomplished current level of school, 1 = drop out of compulsory or secondary school), Maternal unemployment (0 = No long-term unemployment prior to childbirth, 1 = Long-term unemployment prior to childbirth), Parental unemployment (0 = both parents employed, 1 = one or both parents unemployed), Parental profession (0 = white collar, 1 = blue collar/unemployed)

### Single mothers

#### Crosstabs

Among mothers who were single at childbirth, 20.6% experienced symptoms of postpartum depression compared to 11.5% of cohabitating mothers (*p* = 0.028). Mothers who were single at child birth reported externalizing problems in their children at age 3 more often (20.4%) than cohabitating mothers (9.0%) (*p* = 0.005). Likewise, 20.4% of mothers who were single at child birth reported internalizing problems in their children at age 3 compared to 9.2% of cohabitating mothers (*p* = 0.006). No significant differences in children’s CWC scores were found.

#### Logistic regression

Mothers who were single at childbirth were not at increased risk for symptoms of postpartum depression (OR 1.000, CI 0.495–2.020) (Table 3). Likewise, they did not report externalizing or internalizing problems in their children to a greater extent than cohabitating mothers (OR 1.422, CI 0.636–3.183; OR 1.985, CI 0.934-4.217). CWC scores did not differ between children of mothers who were single at child birth compared to children of cohabitating parents. When the effect of cohabitation status on symptoms of postpartum depression was tested, lack of completion of compulsory or secondary school was the most influential factor (OR 3.457, CI 2.269–5.267). Across all scales, parental unemployment had a significant effect, including CWC score (OR 3.205, CI 1.808–5.681).

**Table 3:** Odds ratios for women who were non-cohabitating at childbirth, when predicting depressive symptoms as well as behavior and development in their children at age 3

	Odds Ratio	95.0% CI for OR	*p*-value
EPDS ≥ 10			
Maternal single status	1.000	0.495–2.020	0.999
Maternal education	3.457	2.269–5.267	< 0.001
Maternal unemployment	1.813	1.084–3.032	0.023
Externalizing ≥ 90th percentile			
Maternal single status	1.422	0.636–3.183	0.391
Parental unemployment	2.800	1.763–4.449	< 0.001
Parental profession	1.695	1.107–2.594	0.015
Cohabitation status at follow-up	1.420	0.805–2.506	0.226
Internalizing ≥ 90th percentile			
Maternal single status	1.985	0.934–4.217	0.075
Parental unemployment	2.500	1.557–4.014	< 0.001
Parental Profession	1.949	1.275–2.981	0.002
Cohabitation status at follow-up	1.260	0.703–2.260	0.438
CWC Score ≥ 3			
Maternal single status	1.336	0.477–3.736	0.581
Parental unemployment	3.205	1.808–5.681	< 0.001
Parental profession	1.499	0.865–2-598	0.149
Cohabitation status at follow-up	1.335	0.647–2.755	0.434

Note: *CI* = Confidence Interval, *OR* = Odds ratio, *EPDS* = Edinburgh Postnatal Depression Scale, *CWC* = Child Welfare Center. Logistic regression. Dependent variables: EPDS, CBCL (internalizing, and externalizing) and CWC score. Independent variables (0 is used as a reference level): Maternal cohabitation status at childbirth (0 = cohabiting with the child’s father, 1 = single status or other arrangements), Maternal education (0 = accomplished current level of school, 1 = drop out of compulsory or secondary school), Maternal unemployment (0 = No long-term unemployment prior to childbirth, 1 = Long-term unemployment prior to childbirth), Cohabitation status at follow-up (0 = cohabitating, 1 = single status), Parental unemployment (0 = both parents employed, 1 = one or both parents unemployed), Parental profession (0 = white collar, 1 = blue collar/unemployed)

### Post hoc analysis

Given the association between lack of completion of compulsory or secondary school and symptoms of postpartum depression, the difference in symptoms of postpartum depression between mothers who dropped out of school (*n* = 1450) and those who did not (*n* = 153) was examined. Twenty-nine point four % (*n* = 45) of the mothers who did not complete compulsory or secondary education reported symptoms of postpartum depression compared to 10.6% (*n* = 153) of those who did finish (*p* < 0.001). There were no differences in frequencies of child internalizing and externalizing problems or CWC score between children of mothers who dropped out of school and those who did not.

## Discussion

### Young mothers

In accordance with previous studies, the results indicate that giving birth at a young age is associated with symptoms of postpartum depression. Several factors might be of importance for this finding. Firstly, an adolescent mother faces the challenge of her own developmental tasks besides the challenge of taking care of a newborn. Secondly, early motherhood is associated with lower degrees of education and lower income [22]. As shown in this study, school drop-out was the most influential factor for postpartum depression symptoms. Moreover, adolescent pregnancy has previously been shown to be linked to other factors increasing the risk for mental health problems, factors such as lower family SES and aggressive and delinquent behaviors [23].

Young mothers reported more emotional and behavioral problems in their children, as has been noted previously [2]. However, it has to be taken into consideration that maternal mental health could influence how the mother perceives and reports on behavior in her child, which could explain part of the increased risk for child behavioral and emotional problems. This in turn, could influence parenthood through anxiety or frustration and create a downward spiral for family wellbeing.

No association was found between young maternal age and the physical and mental development of the child as assessed at the routine medical examination at age 3, which could be interpreted as a promising sign. However, as discussed earlier, a detrimental environment during pregnancy and the first years in life when brain development is intense, could have effects on behavior and stress response apparent later in life [24].

### Single mothers

Quite contrary to findings in the majority of previous studies, mothers who were single at childbirth did not report symptoms of postpartum depression to a greater extent than cohabitating mothers. Since the reason for single status of the women in the present study is not known, one can only hypothesize that this group might be more heterogeneous than the divorced women usually included in this type of studies. Living arrangements were not specified in the present study, and it is likely that the mother’s social network is of importance. Likewise, the quality of contact with the father has been shown to impact child wellbeing [25], but was not controlled for in the present study.

Women who were single at childbirth did not report emotional or behavioral problems in their children to a greater extent than cohabitating mothers. Increased rates of depression and anxiety have been shown earlier in school-aged children of divorce [26], however, the separation in itself might be the stress triggering behavioral and emotional problems [27]. Likewise, an association between single motherhood and externalizing problems have previously been reported in older children [12]. Almost 2/3 (*n* = 22) of the women who were single at baseline, reported cohabitation at the 3-year follow-up, which has to be taken into account when interpreting the results. These families are maybe less likely to experience lower SES due to the structural inequalities of one parent households, however, the children might be subjected to a major life event of family restructuring.

### Sociodemographic factors

Low SES is known to be linked to both early and single motherhood. The present study controlled for unemployment, profession, and lack of completion of compulsory or upper secondary school; the latter was shown to be more influential on depressive symptoms than both young age and single status. Just as researchers have argued previously, risk factors for mental health problems tend to coexist due to structural inequalities that tend to reproduce between generations [15]. Health care workers need to be aware of the increased risk for mental health problems especially in young mothers, and to continuously follow up on mental health and development in these families. Societal support for young mothers is needed to enable them to continue their education, but also to support parenthood and wellbeing and facilitate the transition from childhood to adulthood when it involves parenthood.

An important observation is that parental unemployment increased the risk for adverse outcomes in all models, including child development (CWC score). Most likely, mediating factors play a role in this association. Moreover, the relationship could be bidirectional, for example, developmental delays in a child could impact the possibility of parental labor.

### Limitations

First, reports on mental health and child behavior came from the mothers, and no clinical assessment of mental health was obtained The CWC score included behavior, but as only one of five domains where the focus was general development. Previous research has raised concerns about biased reports on child behavior in mothers who suffer from mental health problems [28]. However, others say this does not impact the results to a great extent [29]. Furthermore, one can argue that it is reasonable to believe that the wellbeing of a small child is best known by the primary caregiver.

Despite the use of a large population based cohort, the numbers of mothers in the index groups were small. Thus, the results should be interpreted with caution due to the low number of observations in each combination of variables. Moreover, the young mothers did not participate in the follow-up to the same extent as mothers age 21 and above. This can possibly cause an underestimation of the risks of being a young mother, since the overrepresentation of loss to follow-up was extra prominent in the variables internalizing and externalizing problems. Low income, unemployment and low educational status are factors known to increase the dropout rate in longitudinal studies [30], and from the results of the present study it is obvious that young mothers are a vulnerable group. Likewise, mothers of foreign origin did not participate in the follow-up to the same extent as mothers born in Sweden. While there is support in the literature for similar levels of internalizing and externalizing problems reported by immigrant and non-immigrant mothers [31], immigrants might not dispose the same resources (SES, language, social network etc) as Swedish-born parents. The families who dropped out might thus be burdened and would have made an interesting contribution to the follow-up results. The SESBiC cohort was followed further through childhood, but at age 12 the retention rate was too low to carry out multivariate analyses on sub groups.

Another limitation of the present study is the lack of data on quantity and quality of contact with the child’s father and other family and social network contacts. For a young mother living with her parents, or for a single mother to have the child’s father or other person especially close to the mother, sharing the responsibility for the child, the stress is likely to be reduced. This kind of support is possibly a mediating factor which could affect the outcome of mental health and behavior, and is suggested here as an issue for future studies.

In conclusion, the study is strengthened by the use of a birth cohort and not a specifically burdened population. The aim of the study was not to stigmatize young mothers and single mothers, but to pinpoint associated risks for these families, and in conclusion to illuminate the need for societal support.

## Conclusions

Young mothers are at increased risk for symptoms of postpartum depression which indicates the need for attention in pre- and postnatal health care programs. Single mothers and their children were not found to be at increased risk for adverse outcomes. The importance of schooling was demonstrated, indicating the need for societal support to encourage adolescents to remain in school.

## Abbreviations

CBCL: Child Behaviour Checklist
CI: Confidence Interval
CWC: Child Welfare Center
EPDS: Edinburgh Postnatal Depression Scale
MDD: Major Depressive Disorder
OR: Odds Ratio
SES: Socioeconomic Status
SESBiC: South East Sweden Birth Cohort Study
